# Growth Arrest of BCR-ABL Positive Cells with a Sequence-Specific Polyamide-Chlorambucil Conjugate

**DOI:** 10.1371/journal.pone.0003593

**Published:** 2008-10-31

**Authors:** C. James Chou, Thomas O'Hare, Sophie Lefebvre, David Alvarez, Jeffrey W. Tyner, Christopher A. Eide, Brian J. Druker, Joel M. Gottesfeld

**Affiliations:** 1 Department of Molecular Biology, The Scripps Research Institute, La Jolla, California, United States of America; 2 Oregon Health & Science University Cancer Institute, Division of Hematology and Medical Oncology, Portland, Oregon, United States of America; The Scripps Research Institute, United States of America

## Abstract

Chronic myeloid leukemia (CML) is characterized by the presence of a constitutively active Abl kinase, which is the product of a chimeric BCR-ABL gene, caused by the genetic translocation known as the Philadelphia chromosome. Imatinib, a selective inhibitor of the Bcr-Abl tyrosine kinase, has significantly improved the clinical outcome of patients with CML. However, subsets of patients lose their response to treatment through the emergence of imatinib-resistant cells, and imatinib treatment is less durable for patients with late stage CML. Although alternative Bcr-Abl tyrosine kinase inhibitors have been developed to overcome drug resistance, a cocktail therapy of different kinase inhibitors and additional chemotherapeutics may be needed for complete remission of CML in some cases. Chlorambucil has been used for treatment of B cell chronic lymphocytic leukemia, non-Hodgkin's and Hodgkin's disease. Here we report that a DNA sequence-specific pyrrole-imidazole polyamide-chlorambucil conjugate, 1R-Chl, causes growth arrest of cells harboring both unmutated BCR-ABL and three imatinib resistant strains. 1R-Chl also displays selective toxicities against activated lymphocytes and a high dose tolerance in a murine model.

## Introduction

Chronic myeloid leukemia (CML) is characterized by the presence of a constitutively active chimeric Bcr-Abl kinase. The BCR-ABL chimeric gene arises from a genetic translocation between the ABL proto-oncogene on chromosome 9 and the breakpoint cluster region (BCR) on chromosome 22 [1], [2], [3]. In the late 1990s, imatinib emerged as a single agent treatment for CML, and was approved for first-line treatment of CML. Imatinib has revolutionized treatment for this disease [4]. Patients who received imatinib during the chronic phase have a very good prognosis with survival rate of 89% by 60 months and freedom from progression to accelerated phase (AP) or blast crisis (BC) of 93% [5], [6]. However, imatinib responses are much less durable in patients with AP or BC, and there is no single standard therapy for these stages of CML [7], [8], [9]. Alternative Abl kinase-selective inhibitors have been developed to overcome resistance to imatinib. Two compounds in particular, dasatinib and nilotinib, inhibits 14 of out of the 15 imatinib-resistant BCR-ABL mutants [10], [11]. However, dasatinib and nilotinib do not inhibit the mutant derived from the T315I substitution; thus potential resistant mutations are still possible.

**Figure 1:**
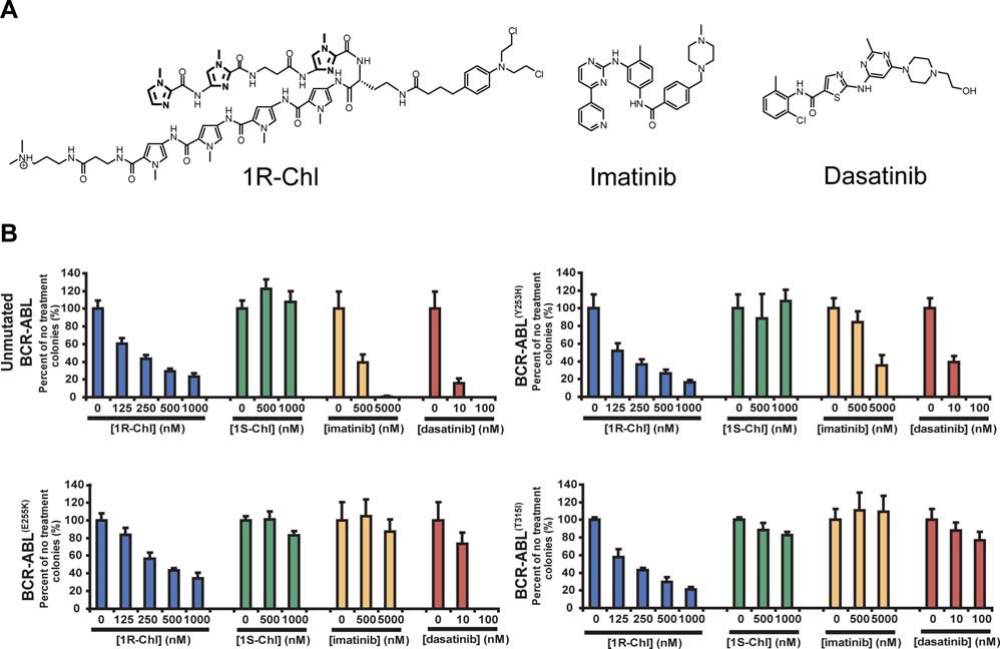
Chemical structure of 1R-Chl, imatinib, dasatinib, and their effects on BCR-ABL unmutated and mutated genes transduced into murine BM cells. (A) 1R-Chl (left) targets the DNA sequences 5′-WGGWGW-3′. Bold, imidazole rings. imatinib (middle) targets Bcr-Abl kinase, and dasatinib (right) targets 14 out of 15 Bcr-Abl mutants. (B) Murine BM cells transduced with unmutated BCR-ABL and single point mutation Y253H, E255K, and T315I genes were tested for the effectiveness of 1R-Chl (125 nM to 1000 nM), 1S-Chl (500 nM and 1000 nM), imatinib (500 nM and 5000 nM; IC_50_ = 260 nM for the native BCR-ABL transduced cells) [6], and dasatinib (10 nM and 100 nM; IC_50_ = 0.8 nM for the native BCR-ABL transduced cells) [6]. Triplicate experiments were done, and the numbers of colonies were quantified 7 days after initial plating.

Chlorambucil (Chl) is an effective first-line single-agent treatment for chronic lymphocytic leukemia and as a combined chemotherapeutic in low-grade non-Hodgkin's and Hodgkins' disease [12], [13]. However, Chl has poor affinity toward its known target DNA, with efficacy only at ∼100 µM concentration [14], [15]. When conjugated to pyrrole-imidazole (Py-Im) polyamides, Chl has shown increased affinity and specificity toward DNA [16], [17], [18]. By screening a small library of polyamide-Chl conjugates, we identified one specific conjugate, termed 1R-Chl (Figure 1A), which blocks proliferation of various cancer cell lines in culture. 1R-Chl has anti-tumor activity in mice bearing human SW620 colon carcinoma, K562 CML, Calu-1 lung cancer and 22Rv1 prostate cancer xenografts [19], [20]. Microarray analysis indicated that limited numbers of genes are significantly down regulated by 1R-Chl. One such gene encodes histone H4 (H4c), and western blotting experiments confirmed that total H4 protein levels are reduced in 1R-Chl treated cells. 1R-Chl is expected to bind the general DNA sequence 5′-WGGWGW-3′, where W = A or T., This sequence is found in the histone H4 gene, which is bound by the parent compound lacking Chl and alkylated by 1R-Chl. Alkylation within the coding region of the H4c gene was confirmed in cell culture by ligation-mediated PCR. Such alkylation leads to down-regulation of H4 gene expression, with a concomitant loss of H4 protein. This leads to nucleosome disruption, widespread alkylation within genomic DNA and cell cycle arrest at G2/M. Changing the chirality of the “turn” amino acid to S-2,4-diaminobutyric acid (1S-Chl) eliminates the biological activity of the conjugate due to a loss of binding and alkylation of this target DNA sequence. 1R-Chl has also been shown to inhibit proliferation of the CML cell line K562 at nanomolar concentrations [20]. Most importantly, polyamide-Chl conjugates that do not target the H4c gene fail to down-regulate this gene or block cell proliferation [19], [20].

In this study, we investigated the effects of 1R-Chl, and in combination with imatinib, on native and mutated BCR-ABL transduced murine bone marrow (BM) cells. Studies were also done using primary murine BM cells and mononuclear cells from normal donors and CML patients. In addition, dosing experiments of 1R-Chl have been examined in the murine model. Our results suggest that 1R-Chl is effective in blocking proliferation of cells harboring most of the common mutations found in Bcr-Abl kinases. 1R-Chl also works additively with imatinib in murine BM cells transduced with BCR-ABL. 1R-Chl only displayed growth inhibition activity and toxicity against proliferating cells, while no toxicity was found in non-cycling cells. In addition, 1R-Chl displayed a high dose tolerance in the murine model, with liver toxicity not observed until levels approaching ten times the therapeutic dose were reached.

## Results

### Polyamide-Chl conjugates

For this study, we used 1R-Chl, ImIm-β-Im-α(*R*-2,4-DABA^Chl^)-PyPyPyPy-β-Dp (where Py is pyrrole, Im is imidazole, β is β-alanine, Dp is dimethylaminopropylamine, and 2,4-DABA is *R*-2,4-diaminobutyric acid, with α describing the amino acid linking the hairpin polyamide and Chl describing the chlorambucil substation at the 4-amino position), and its inactive stereoisomer 1S-Chl, where the turn amino acid is -α(*S*-2,4-DABA) (Figure 1A). These compounds have been extensively characterized previously [20], [21], [22].

### 1R-Chl inhibits the growth of bone marrow cells transduced with BCR-ABL or Y253H, E255K, and T315I BCR-ABL mutants

Murine bone marrow cells that were transduced with full-length native BCR-ABL, or BCR-ABL with kinase domain Y253H, E255K, and T315I point mutations, were treated with different concentrations of 1R-Chl, 1S-Chl, imatinib, and dasatinib (Fig. 1B). 1R-Chl showed titratable inhibition of murine BM colony formation for the unmutated Bcr-Abl and all kinase domain point mutations studied here. The IC_50_ for blocking cell proliferation is approximately 250 nM, similar to the IC_50_ for imatinib. The control polyamide 1S-Chl, the stereoisomer of 1R-Chl, as expected, did not display any inhibitory activity up to 1000 nM.

### 1R-Chl inhibits colony formation by CML patient MNCs and normal human MNCs

**Figure 2:**
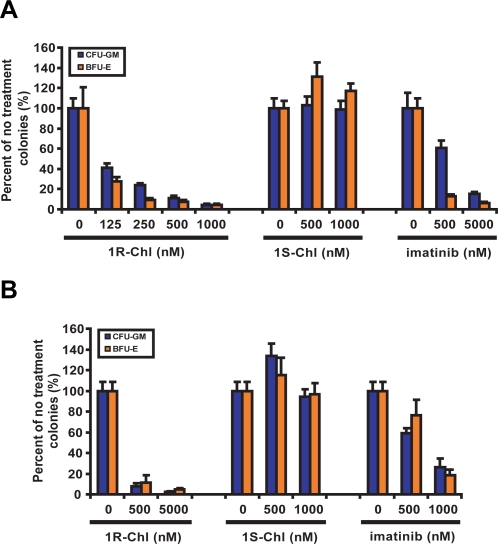
Effects of 1R-Chl on colony formation of CML patient MNCs and normal human MNCs. (A) MNCs from CML patients were cultured with rh stem cell factor, rh IL-3, rh GM-CSF with or without erythropoietin which lead to CFU-GM or BFU-E cell lineages. The experiments were done in duplicate with 1R-Chl ranging from 125 to 1000 nM, 1S-Chl at 500 and 1000 nM, and imatinib at 500 and 5000 nM. The colonies were counted and results were calculated as the percentage of the control plates (without treatment) after 2 weeks. (B) MNCs from normal donors were cultured and plated under the same condition as CML patient cells. The cells were exposed to 500 and 1000 nM 1R-Chl, 500 and 1000 nM 1S-Chl, and 500 and 5000 nM imatinib. The colonies were counted and percent growth inhibition was calculated after 2 weeks.

1R-Chl, 1S-Chl, and imatinib were tested against bone marrow mononuclear cells from patients in chronic phase of CML. The cells were cultured in semisolid media containing different growth factors (rh stem cell factor, rh IL-3, rh GM-CSF with or without erythropoietin). Cells were exposed to 1R-Chl, 1S-Chl, and imatinib at different concentrations (Fig. 2A). The results show that exposing CML patient MNCs to 1R-Chl suppressed colony formation in both CFU-GM and BFU-E assays, while 1S-Chl was ineffective. Thus, 1R-Chl was effective against CML patient MNCs with an observed IC_50_ less than 125 nM, and in the murine BM experiments (Fig. 1). Potential non-specific toxicity of 1R-Chl was observed with normal human MNCs (Fig. 2B); however, such toxicity may be explained by the unusually high effectiveness of 1R-Chl against activated human MNCs.

### Additive effects of 1R-Chl in combination with imatinib

**Figure 3:**
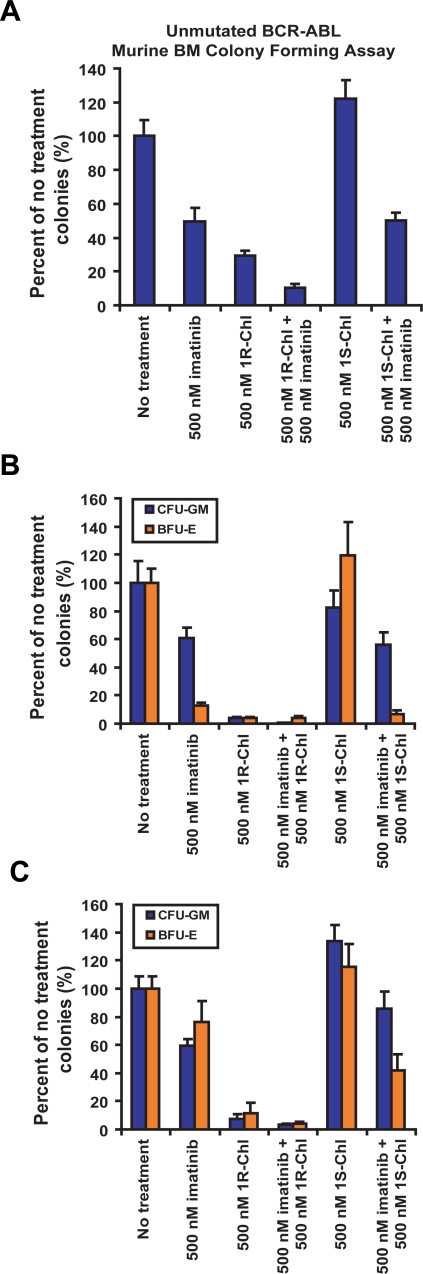
Additive effects of 1R-Chl and imatinib treatments. (A) Murine BM cells transduced with unmutated BCR-ABL were treated with 500 nM imatinib, 500 nM 1R-Chl, and 500 nM 1S-Chl individually, or in combination of either 500 nM 1R-Chl or 500 nM 1S-Chl with 500 nM imatinib. (B) Same treatment routines were used on CML patient MNCs. The CML patient MNCs were treated 500 nM imatinib, 500 nM 1R-Chl, and 500 nM 1S-Chl individually, or 500 nM 1R(S)-Chl in combination with 500 nM imatinib. (C) Same experiments were done on normal blood donor MNCs.

The additive effects of 1R-Chl and imatinib were also studied in BCR-ABL transduced murine BM, CML patient MNCs, and normal human donor MNCs. Inhibition of colony formation by 1R-Chl and imatinib was additive in unmutated BCR-ABL-expressing murine BM (Fig. 3A). In MNCs from CML patients and normal blood donors, 1R-Chl was a stronger growth inhibitor than imatinib, and it appeared that 1R-Chl treatment alone at 500 nM is sufficient to completely (>90%) inhibit colony formation by these cells (Fig. 3B and 3C). The observed additive effects of 1R-Chl and imatinib indicate that a cocktail of 1R-Chl with imatinib could potentially increase the efficacy of imatinib treatment and prevent the emergence of imatinib-resistant CML cells. However, the strong colony formation inhibition of normal human MNCs by 1R-Chl could potentially indicate a non-specific toxicity response due to 1R-Chl. Further experiments on both murine BM and normal human donor MNCs were done to address this issue.

### No significant cell toxicity observed for the resting murine BM cells and human MNCs

**Figure 4:**
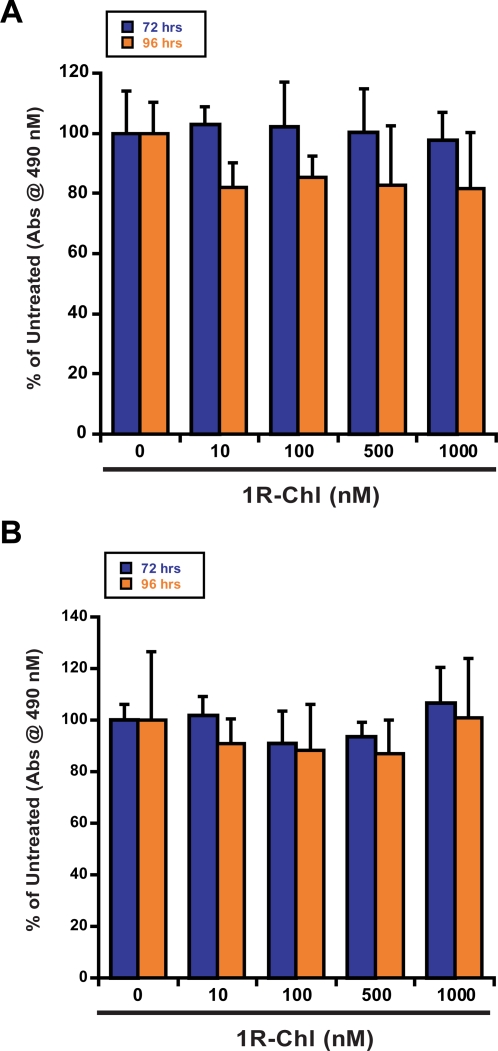
Cytotoxicity (MTS) assays on resting murine BM cells and human MNCs. (A) Ficoll-Hypaque-separated murine BM cells were plated in triplicate with 1R-Chl (10, 100, 500, and 1000 nM) without any growth factors or mitogens. After 72 and 96 hours, 20 µL of Celltiter 96 Aqueous One solution (Promega, WI) was added. The absorbances of the MTS metabolites were read corresponding to the numbers of metabolically active cells. (B) Ficoll-Hypaque-separated human MNCs were treated as above, and the viabilities of the cells were assessed after 72 and 96 hours.

To determine if the non-specific toxicity of 1R-Chl described above was due to the activation of mononuclear cells, the following assays were performed. Murine BM cells and human peripheral blood lymphocytes (hPBLs) were harvested and treated with different concentrations of 1R-Chl, without activation by growth factors or mitogens. MTS (3-(4,5-dimethylthiazol-2-yl)-5-(3-carboxymethoxyphenyl)-2-(4-sulfonphenyl-2H-tetrazolium salt) cytotoxicity assays (Promega, WI), which examine the mitochondrial activity of cells, were used to examine the metabolic state of the cells. MTS is bio-reduced by the mitochondria into a colored formazan product. This conversion is only accomplished by the dehydrogenase enzymes in metabolically active cells. For either resting murine BM or normal human donor MNCs, there is no significant cytotoxicity observed after treatment with 1R-Chl at concentrations ranging from 10 to 1000 nM, after incubation for 72 or 96 hours (Fig. 4A and 4B). These data indicate that 1R-Chl does not exhibit toxicity against non-proliferating murine BM cells and human MNCs, and the colony forming inhibition was probably due to growth inhibition of proliferative cells.

### 1R-Chl is well tolerated in mice

**Figure 5:**
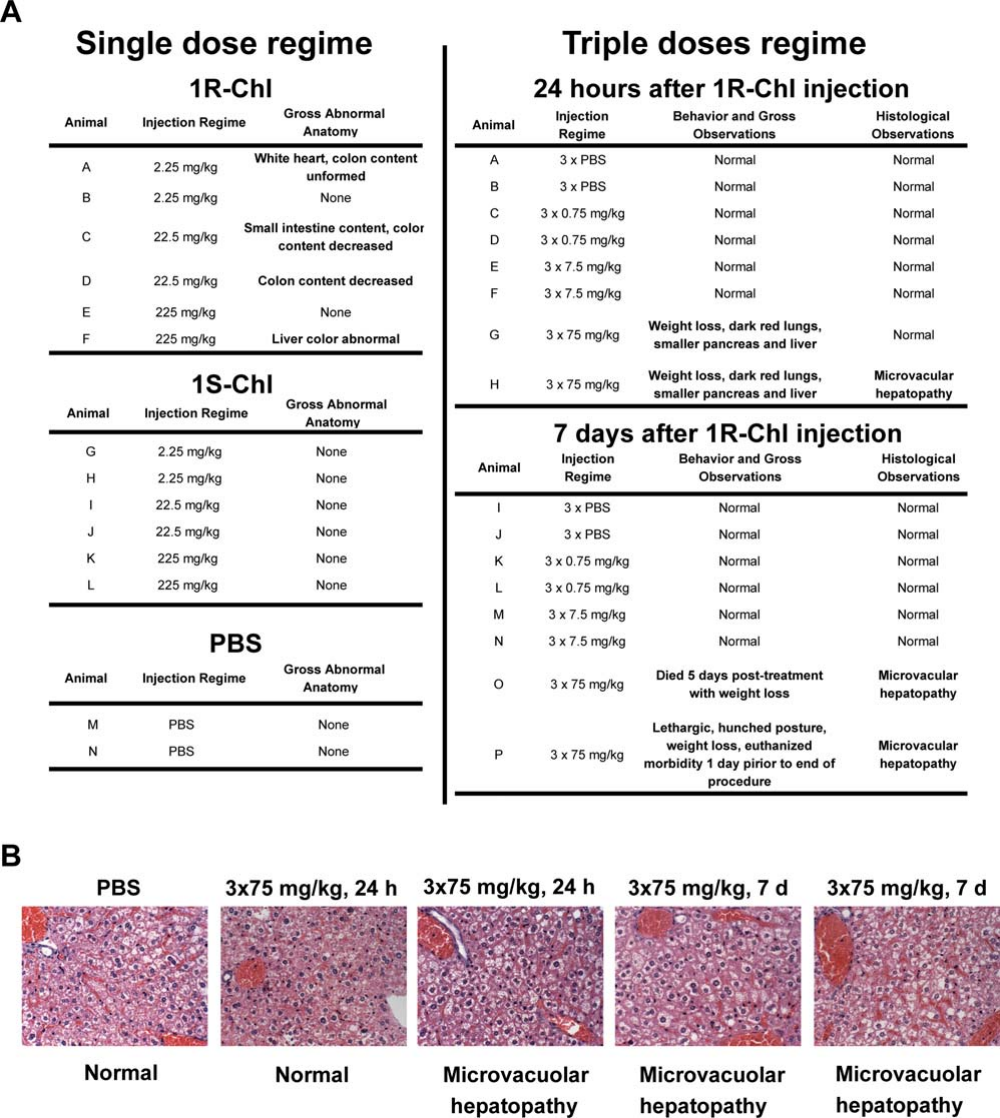
Pharmacotoxicity of 1R-Chl and 1S-Chl in mice. (A) After single I.V. bolus injection of 1R-Chl at 2.25, 22.5, or 225 mg/kg, and PBS into Balb/c mice, the mice were subjected to gross anatomical observation followed by detailed histological analysis (Left). Mice were also injected with 1R-Chl with the indicated dose (0.75, 7.5, and 75 mg/kg) three times in one week. Half of the experimental population was euthanized after 24 h, and the other half after 7 days (as indicated). Animals were subjected to gross behavioral and anatomical observation followed by a detailed histological analysis (Right). (B) Toxic doses of 1R-Chl can cause microvacuolar hepatopathy. Mice were subjected to the toxicity studies, including injections of a dose-regime ten times higher than the therapeutic dose-regime. Representative tissues, including liver, were fixed in 10% formalin, trimmed for histology, embedded in paraffin, 3.0 micron sections were cut, mounted on glass slides and stained with H&E. Photomicrographs of H&E sections from liver of animals that received PBS and euthanized 7 days post-treatments (control), 3×75 mg/kg and euthanized 24 h post-treatment, and 3×75 mg/kg and euthanized 7 days post-treatments were shown (indicated above each picture). Morphology of the section is indicated below each picture.

The potential toxicity of 1R-Chl *in vivo* was examined by I.V. injections in Balb/c mice. For the short-term dosing experiment, mice given 3 single-dose injections at 2.25 mg/kg, 22.5 mg/kg or 225 mg/kg, were monitored for 24 hours. For long-term dosing experiments, 3 lower doses of 1R-Chl were given at 0.75 mg/kg, 7.5 mg/kg, and 75 mg/kg, 3 times every other day for one week. As summarized in Fig. 5A, only the regimen involving three injections of 75 mg/kg 1R-Chl over one week was toxic, leading to death 3–5 days after the last injection. The short-term experimental mice showed no obvious signs of toxicity; however, there were some inconsistent histology results, and most animals were essentially normal, except for the four mice in the 75 mg/kg regimen. The two animals that died prior to the end of the treatment had pale, light red/brown livers. In addition, both the livers and the spleens of all four of the 75 mg/kg 1R-Chl-treated mice were small compared with either the low-dose 1R-Chl-treated animals or PBS-recipients. Microscopically, the livers from three of the four highest-dose animals had microvacuolar hepatopathy (Fig. 5B), consistent with Reye-type syndrome of Balb/c mice. However, and most importantly, at 7.5 mg/kg treatment doses, where 1R-Chl completely inhibits xenograft growth, no weight loss nor abnormal organ and tissue histology were observed in the mice [19], [20].

## Discussion

We have previously shown that 1R-Chl exerts its anti-proliferative effects on cultured cells, and in mouse xenograft models for human cancer, through a two-hit mechanism: 1R-Chl binds and alkylates members of the histone H4 gene family, and causes down-regulation of histone gene transcription [19], [20], [23]. Depletion of histone protein, and concomitant loss of nucleosomes, results in opening of the genome for wide-spread alkylation by 1R-Chl, leading to cell cycle arrest [23]. Thus, the mechanism of action of 1R-Chl is well established. In this study, we investigated treatment of unmutated BCR-ABL and mutated BCR-ABL transduced murine cells as well as primary CML cells from patients with 1R-Chl alone or in combination with imatinib. Previous studies have shown that growth inhibition of the CML cell line K562 by 1R-Chl is independent of Bcr-Abl kinase pathways [20]. Thus, the initial focus of this study was to test the efficacy of 1R-Chl against unmutated BCR-ABL and three BCR-ABL mutants. 1R-Chl has broad range inhibitory activity against BCR-ABL positive cells and the three mutants under investigation. Of particular interest, 1R-Chl shows comparable anti-proliferative activity on BM cells transduced with the T315I mutant, which is resistant to the imatinib and dasatinib treatment, compared to unmutated BCR-ABL. This result is somewhat expected because 1R-Chl inhibits leukemia growth through the down-regulation of the histone H4 transcription and its protein products [19], [20]. We have argued previously that 1R-Chl exerts its anti-proliferative activity through a two-hit mechanism, wherein the primary effect of the compound is to alkylate and down-regulate expression of particular histone genes, including H4c and H4j/k in CML cells [23]. Loss of histone protein leads to nucleosome loss, genome opening, widespread DNA alkylation and subsequent cell cycle arrest. None of these pathways are known to involve the Bcr-Abl kinase.

Additive effects of the two drugs have been observed when 1R-Chl is used in combination with imatinib. In murine cells transduced with the BCR-ABL fusion gene, the effects of 1R-Chl and imatinib on cell growth inhibition are additive and are more effective than either single drug treatment. The combined effects of 1R-Chl and imatinib are harder to judge in the primary cell assays due to the observed increase in potency of 1R-Chl against primary cells. However, there still appears to be additivity when the combined treatments were used. These results indicate a cocktail treatment of 1R-Chl in combination with imatinib could potentially be an effective treatment for CML and prevent or delay drug resistance.

The inhibitory potency of 1R-Chl has also been tested on primary cells from CML patient MNCs and normal blood donors. Titratable growth inhibition is observed in the CML patient MNCs treatments while the control polyamide 1S-Chl is ineffective. Throughout this investigation, it also appeared that 1R-Chl is a stronger inhibitor against MNCs from CML patients (IC_50_<125 nM) than transduced murine cells (IC_50_<250 nM). One alarming aspect, however, is that 1R-Chl also blocks the proliferation of stimulated normal human donor MNCs. To address the possibility of non-specific toxicity, 1R-Chl has been tested against resting primary cells from both human and murine sources. 1R-Chl only inhibited cell growth of proliferating cells and no non-specific toxicity was observed in resting primary cells, as judged by MTS assays for mitochondrial function. When cells are not proliferating, 1R-Chl has little effect, which is consistent with the proposed mechanism of action of 1R-Chl (through down-regulation of H4 genes, thus inhibiting cell division).

To further investigate potential toxicity of 1R-Chl, murine dosing experiments were performed on normal BALB/cByJ mice, and they have shown surprisingly high dose tolerance for 1R-Chl. All mice survived 24 hours post injection, even at 10 times the normal treatment doses (225 mg/kg single dosing or 75 mg/kg at three injections in one week). With the exception of mice that received the highest doses (75 mg/kg), no obvious changes or abnormalities in organ appearance were noted. This is consistent with previous xenograft experiments (SW620 and K562 cell lines), in which athymic nude mice were treated at (7.5 mg/kg per dose) three doses every other day over 1 week via tail vein injections [19], [20]. Only those mice that received high doses (a 225 mg/kg single dose or three doses of 75 mg/kg over 1 week) showed liver anomalies and microvacuolar hepatopathy on microscopic observation of liver slices. With the exception of the high dose animals, there was also no significant weight loss. In previous studies, a diastereomer of 1R-Chl (where the hairpin turn amino acid is γ-diaminobutyric acid), targeting the same sequence as 1R-Chl but unable to discriminate single base pair mismatches, is lethal under the same treatment conditions within 24 hours while 1R-Chl treated mice maintained their weight and behave normally. These data indicate, at least in the small animal model, 1R-Chl unlike other potent DNA alkylating agents is very well tolerated [19], [20], [21], [24], [25].

Selective Bcr-Abl tyrosine kinase inhibitor imatinib (STI 571, Gleevec, Novartis) as the frontline therapeutic for CML has been a major advance in cancer therapy [26], [27]. However, many resistance mutations to imatinib and the acquisition of Bcr-Abl-independent genetic abnormalities during the course of treatment have been reported [28], [29], [30], [31], [32]. Thus, development of additional therapeutic approaches for CML and other malignancies is worthwhile. 1R-Chl is capable of inhibiting K562 CML cell growth both *in vitro* and *in vivo*. 1R-Chl is also highly effective in blocking K562 xenograft growth, with high dose tolerance in the murine model. Polyamide-Chl conjugates appear to be promising cancer therapeutics. In addition, the inhibitory mechanism of 1R-Chl in K562 cells is likely to be independent of BCR-ABL because no significant down-regulation of BCR-ABL transcripts was observed in either Affymetrix genechip analysis or real time qRT-PCR [20]. Based on the findings of this study, these results suggest 1R-Chl could be very effective against CML alone or in combination with imatinib. Also, 1R-Chl is very effective against imatinib resistant Bcr-Abl mutants including T315I, which is resistant to all other Bcr-Abl kinase inhibitors including dasatinib. The overall chemotherapeutic treatments are more effective when 1R-Chl and imatinib are used in combination and the effects appeared to be additive. Most importantly, 1R-Chl is the first sequence specific DNA alkylator reported to show little animal toxicity at mid to high mg/kg range with 3 consecutive injections every other day over 5 days. No behavior or significant weight changes have been observed during normal Balb/c and xenograft mice experiments [19], [20], [21]. Tallimustine and Brostallicin, micromolar DNA alkylators targeting AT rich sequences, have both reported significant animal toxicity at 4 mg/kg and 0.8 mg/kg respectively [24], [25]. These results suggest that the use of 1R-Chl in combination with imatinib and other kinase inhibitors or anti-cancer chemotherapeutics could potentially overcome the potential resistance issues in CML.

## Materials and Methods

### Reagents

1R-Chl and 1S-Chl were synthesized at the Scripps Research Institute and the California Institute of Technology as previously described [16], [21]. Imatinib was purchased from the Oregon Health & Science University pharmacy. 1R-Chl and 1S-Chl were prepared as 1 mmol/L stock solutions in water, and imatinib was prepared as a 10 mmol/L stock in PBS. Experiments were performed with dilutions of the initial stock solutions.

### Murine BM cell colony-forming assays

To examine the efficacy of 1R-Chl against BCR-ABL transduced primary cells, murine BM cells were pre-stimulated with IL-3, IL-6, and SCF. The BM cells were than transduced twice with 1×10^6^ plaque-forming units of murine stem cell retrovirus (MSCV) expressing native or mutant BCR-ABL (E255K, Y253H, and T315I) [33]. The cells were then plated in Methocult (5×10^4^ per plate) without cytokines in the absence or presence of 1R-Chl (125 to 1000 nM), 1S-Chl (500 and 1000 nM), and imatinib (500 and 5000 nM), and dasatinib (10 and 100 nM). The numbers of colonies were quantified 7 days after plating.

### CML patient and normal donor mononuclear cell (MNCs) colony forming assays

Colony-forming assays were performed on primary cells from CML patients and normal donor blood. The cells were incubated in the presence of recombinant human (rh) stem cell factor (50 ng/mL), 10 ng/mL rh GM-CSF, and 10 ng/mL rh interleukin-3 (IL-3) for assessment of granulocyte-macrophage colony-forming unit (CFU-GM) cells or with erythropoietin for assessment of blast forming unit erythroid (BFE-E). In brief, Ficoll-Hypaque-separated cells were plated in duplicate in methylcellulose medium with cytokines as described (IL-3, GM-CSF, SCF, and +/−EPO) at 5×10^4^/mL with different concentration of 1R-Chl, 1S-Chl, and imatinib. After 2-week incubation at 37°C, BFU-E and CFU-GM colonies were counted and results were expressed as the percentage compared to the number of colonies on control plates.

### Cell viability assays on resting murine BM cells and human blood mononuclear cells

Murine BM cells and human MNCs were plated at 50,000 cells per 100 µL. The cells were then treated with 1R-Chl at 10, 100, 500, and 1000 nM, under standard conditions (RPMI 1640 with 20% FBS) without stimulation by growth factors or mitogens. After 72 and 96 hours, 20 µL Celltiter 96 Aqueous One Solution Cell Assay (Promega, WI) was added. After a 4 hour incubation period, the absorbance was read at 490 nm on a Tecan Infinite 200 plate reader (Tecan Systems Inc. San Jose, CA).

### Pharmacotoxicity of 1R-Chl in mice

1R-Chl toxicities were also examined by I.V. tail vein injections of 1R-Chl at three concentrations followed by either a short-term or long-term experimental protocol. For the short-term studies, the mice were given single-dose injections at 2.25 mg/kg, 22.5 mg/kg or 225 mg/kg via tail vein. For the long-term experiments, 3 lower doses of 1R-Chl were given at 0.75 mg/kg, 7.5 mg/kg, and 75 mg/kg, 3 times every other day throughout one week via tail vein. The 7.5 mg/kg per-injection, 3 times every other day for one week, was the effective treatment routine for previous xenograft studies [19], [20]. For all studies, the behavior and weight of the mice were monitored, and the histology of major organs and tissues was analyzed 24 hours after the last injection. For the long-term experiments, the histology was done either 24 hours after or one week after the last injection.
